# Observations of internal flow inside an evaporating nanofluid sessile droplet in the presence of an entrapped air bubble

**DOI:** 10.1038/srep32767

**Published:** 2016-09-12

**Authors:** Dong Hwan Shin, Jeffrey S. Allen, Seong Hyuk Lee, Chang Kyoung Choi

**Affiliations:** 1 Mechanical Engineering-Engineering Mechanics, Michigan Technological University, Houghton, MI 49931-1295, USA; 2 School of Mechanical Engineering, Chung-Ang University, Seoul 06974, Korea; 3 Center of Urban Energy System Research, Korea Institute of Science and Technology, Seoul, 02792, Korea

## Abstract

Using a unique, near-field microscopy technique, fringe patterns and nanoparticle motions are visualized immediately following a nanofluid droplet deposition on a glass substrate in which an air bubble is entrapped. The nanofluid consists of DI-water, 0.10% Aluminum Oxide nanoparticles with an average diameter of 50 nm, and 0.0005% yellow-green polystyrene fluorescent particles of 1 μm diameter. High-speed, fluorescent-mode confocal imaging enables investigation of depth-wise sectioned particle movements in the nanofluid droplet inside which a bubble is entrapped. The static contact angle is increased when a bubble is applied. In the presence of the bubble in the droplet, the observed flow toward the center of the droplet is opposite to the flow observed in a droplet without the bubble. When the bubble is present, the evaporation process is retarded. Also, random motion is observed in the contact line region instead of the typical evaporation-driven flow toward the droplet edge. Once the bubble bursts, however, the total evaporation time decreases due to the change in the contact line characteristics. Moreover, the area of fringe patterns beneath the bubble increases with time. Discussed herein is a unique internal flow that has not been observed in nanofluid droplet evaporation.

Evaporation of a droplet on a solid surface is applicable to many areas such as inkjet printing, dye painting, and micro-/nano-patterning^1^,^2^,^3^,^4^,^5^,^6^. Furthermore, it is important to understand droplet impact or deposition behavior on the solid substrate because it could affect the evaporation process by changing initial conditions; i.e., contact angle, contact area, and center-height of a droplet. The surrounding gas between the droplet and the solid surface may be trapped when the droplet impacts and deposits on the solid surface. Also, the ambient pressure can be a crucial parameter on the droplet deposition process^7^,^8^,^9^,^10^. Recent studies have reported bubble entrapment inside the droplet when the liquid drop is deposited on the solid surfaces^11^,^12^,^13^,^14^,^15^. Once an air bubble is entrapped, certain defects occur in the above applications. For example, the nanoparticle deposition may become non-uniform and the time required for drying is altered. Furthermore, if the droplets are deposited on a heated surface then the entrapped gas bubble could act as an initiator of nucleate boiling with lower superheat when compared to theoretical predictions^16^,^17^.

There are some of representative internal flows inside an evaporating droplet; evaporation-driven flow, Marangoni flow and buoyancy-driven flow^3^,^18^,^19^. Evaporation-driven flow generates an internal flow into the contact line to replenish liquid that has evaporated^20^. Marangoni flow generates a surface flow due to a temperature gradient between the bottom and top of a droplet and the flow direction is from hot-to-cold along the liquid-gas interface. The density gradient, for example evaporative cooling, generates the buoyancy-driven Rayleigh-Bénard convection caused by temperature and/or concentration difference. The buoyancy flow inside the droplet increases with the Rayleigh number, and buoyancy forces in water droplets on hydrophobic or super-hydrophobic surfaces become dominate^21^. However, for this study all surfaces are hydrophilic, therefore buoyancy-driven flow can be neglected. Thus, this study only focuses on evaporation-driven flow. Figure 1 shows the geometrical shape difference due to entrapment of an air bubble. Once the air bubble is entrapped, the contact angle becomes larger and the droplet contact diameter and center-height increases. When an entrapped air bubble is present the internal flow direction is altered. The change in internal flow direction consequently affects the evaporation process as well as the nanoparticle deposition pattern once the droplets have dried. This study shows the unique internal flow beneath the air bubble and no directional flow (random motion) at the edge of the droplet via the use of an anomalous bubble inside the nanofluid droplet.

## Experimental Method

The working fluid is de-ionized (DI) water with 0.10% of Aluminum Oxide (Al_2_O_3_) nanoparticles (Sigma Aldrich Co.) that have 50 nm average diameters (by volume fraction). The fluid also was seeded with 0.0005% by volume of 1 μm yellow-green polystyrene fluorescent nanospheres (Thermo Fisher Scientific Inc., Invitrogen^TM^, 505 nm (excitation), 515 nm (emission)). The nanofluid was well-dispersed for 5 hours at room temperature and atmospheric pressure by using the ultrasonic disruptor. Droplets were dispensed via a micropipette (Gilson, P2 (F144801) and P10 (F144802)) onto a borosilicate cover slip (DURAN GROUP-Mainz, 24 × 60 mm). The volume of nanofluid droplet applied was 2 μl and 2 μl of air bubble was injected. A gas bubble was placed inside the droplet by injecting air using a micropipette. The equilibrium static contact angle was measured *θ* = 67 ± 0.75° for a droplet without an air bubble, while it was observed *θ* = 89 ± 0.10° for a droplet with an air bubble using a contact angle measurement apparatus developed at Michigan Technological University^22^. The environmental temperature and humidity were maintained at 20 ± 1 °C and 10 ± 2%, respectively.

**Figure 2:**
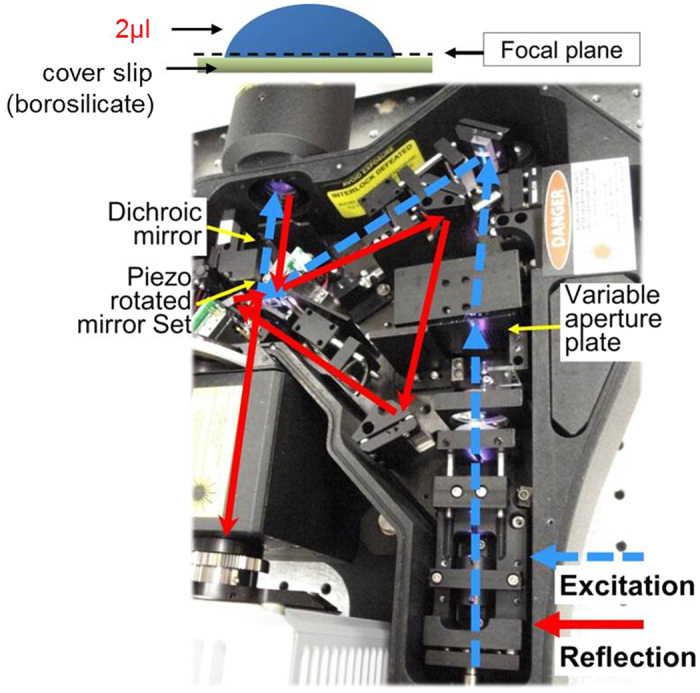
Experimental apparatus: confocal microscope system with light paths. The blue dashed line represents the excitation light path and the red line indicates the reflected light path.

The flow patterns within the droplets were observed using a modified swept-field confocal unit (Prairie Technologies) attached to an inverted compound microscope (Nikon TE-2000) with a precision motorized stage (Prior Scientific ProScan II). The vertical resolution of the stage is ±2 nm, but the accuracy of the stage is ±5 nm. The confocal unit has been modified to operate in either the traditional fluorescence mode or in reflectance mode^23^. For these studies, the confocal was operated in the fluorescence mode. A 488 nm Aurora solid state diode laser was used for illumination. The microscopy setup enabled visualization of particle motions in an optical slice as well as interface shapes via fringe patterns^24^ as shown in Fig. 2. The corresponding confocal optical slicing thickness is approximately 2.3 μm for 40× magnification and 4.3 μm for 20× magnification. The location of the focal plane was determined by first identifying the substrate-liquid interface location based on the peak reflectance intensity and then by moving the stage to the desired focal plane. For the majority of the results discussed, the focal plane was set at 500 ± 5 nm from the top of the substrate, which when using 40× magnification resulted in an optical thickness of 1.65 μm that spanned from the substrate into the liquid. Information above this thickness is spatially filtered using confocal microscopy. Images were captured at 1 frame per second (fps) using a 12-bit CCD camera (JAI CM-141MCL) connected to cameralink frame grabber (EPIX EL1DB). Images were processed using ImageJ^25^.

As stated in the introduction section, a gas bubble can often be entrapped inside a droplet during a deposition process^11^,^12^,^13^,^14^,^15^. Liquid properties, such as surface tension and composition, dictate how long a gas bubble will remain stable within the droplet. In order to have sufficient time to investigate the role of the gas bubble on internal flow during evaporation, liquid properties were varied. The properties varied include nanoparticle concentration (Al_2_O_3_), surface tension (through addition of surfactants), liquid viscosity (through addition of glycerol), and droplet volume. Table 1 lists the various combinations and the resulting observation of gas bubble stability within the droplet. An ‘unstable’ condition is defined by an immediate collapse of the injected bubble into the water droplet while ‘stable’ indicates that the bubble maintains its shape for a certain period of time. When using DI-water, the liquid surface entrapping the bubble ruptured immediately when injecting air. The addition of the aluminum oxide nanoparticles was found to stabilize the air bubble long enough to conduct the experiments when the volume fraction of the nanoparticles was 0.10% and the volume ratio of liquid to air was between 1 and 2.5. Volume fractions of aluminum oxide nanoparticles at 0.01% and 0.05% did not stabilize the air bubble. When the liquid-to-air volume ratio exceeded 2.5 (liquid volume of 5 μl) the air bubble was generally unstable most likely due to the decrease in curvature of the liquid drop. The relationship between drop size, interface curvature, and bubble stability was not the focus of this study and requires further investigation. A surfactant (Span^®^ 80, Sigma Aldrich Co.) was added to the DI-water to investigate the possibility of the aluminum oxide particles might have been contaminated with a surfactant. The total number of gmols of aluminum oxide (MW = 101.96 g/mol) per ml of DI-water was matched with the surfactant (MW = 428.62 g/mol). Thus, approximately 1.5 g of surfactant was added to 100 ml of DI-water. The addition of the surfactant at this concentration did not stabilize the air bubble. Finally, the effect of viscosity on the bubble stability was investigated through the addition of glycerol to the DI-water. The air bubble remained unstable at 10% and 30% mass ratios of glycerol-to-water. Based on these results, the experiments were conducted using 2 μl water droplets with 0.10 vol.% of 50 nm Al_2_O_3_ particles and a 2 μl air bubble.

## Observations and Discussion

**Figure 3:**
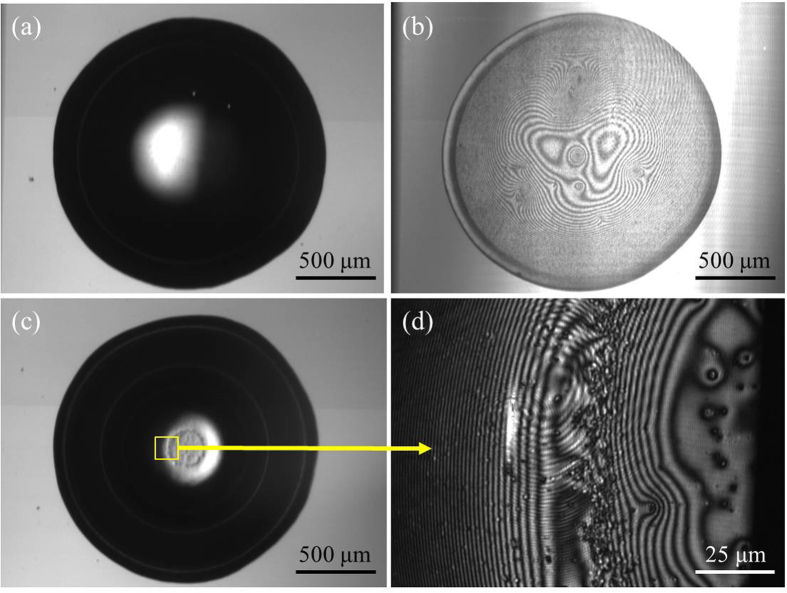
Confocal imaging through the substrate into the droplet (**a**) 2× magnification without air bubble immediately following drop deposition; (**b**) 2× magnification near end of droplet evaporation; (**c**) 2× magnification with air bubble immediately after air bubble injection; (**d**) 40× magnification at 500 nm above the substrate with an air bubble immediately after air bubble injection.

Confocal imaging allows for visualizing velocities in a thin optical plane while simultaneously capturing fringe patterns that describe the shape of the interface. This combination enables precise location of the field of view relative to the contact line and to the air-liquid interface internal to the droplet. At 40× magnification, the confocal plane thickness is 2.3 μm. At 2× magnification, the confocal plane thickness is increased to 257.4 μm. For the results discussed herein, the optical plane location is kept at 500 nm above the substrate except the case where there is depth-wise sectioning imaging. The location and shape of the liquid-air interface created by the presence of the bubble in the liquid drop can be determined from the fringe patterns as illustrated in Fig. 3. Images 2(a) and 2(b) are of a 2 μl droplet without an air bubble at a 2× magnification. Fringe patterns only appear near the end of evaporation when a thin liquid film remains. Images 2(c) and 2(d) are of a 2 μl droplet with a 2 μl air bubble injected at 2× and 40× magnification. Both images are taken at immediately following injection of the air bubble. The location and shape of the air bubble can be seen via the fringe formation in the center of the droplet.

**Figure 4:**
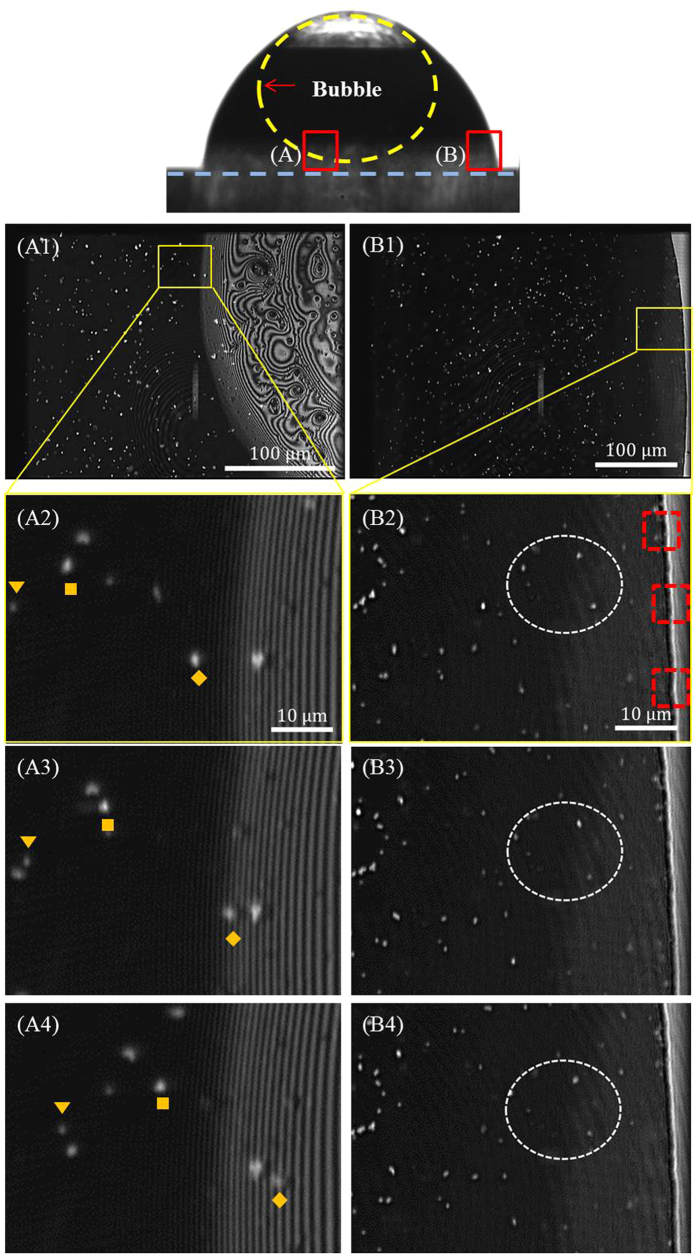
Motion of three particles near (A) center of droplet (beneath the bubble) – motion is from left to right and (B) near the contact line region – motion is random. Images A1 and B1 are the original images. The subsequent sequences of images (A2–A4 and B2–B4) are at 4-second intervals. The dashed rectangle shown in (B2) is the measured sections of intensity variation appearing in Fig. 6(a).

When a droplet evaporates on a solid surface, the evaporation-driven flow moves liquid from the bulk fluid into the contact line region along the substrate in order to replenish liquid that has evaporated in the contact line region. In the location at 500 nm above the substrate in the center of the droplet, there is no directional flow. The random motion of the particles was detected only under the microscope. When the gas bubble is present, the flow into the contact line region appears suppressed and flow is observed moving radially inward at the center of the droplet below the air bubble. Figure 4 illustrates this observation when the air bubble is present. Images from two locations at 40× magnification are shown. Location (A) is near the center of the droplet and location (B) is near the contact line region. The image sequences (A2, A3, A4 and B2, B3, B4) are taken at 4 second intervals. In location (A) near the droplet center, the fluorescent particles are observed moving radially inward between 1 μm/s and 10 μm/s, roughly. In location (A) near the droplet center, the fluorescent particles are observed moving radially inward between 1 μm/s and 10 μm/s. For accurate estimation of particle velocity, the tracking-fluorescence correlation spectroscopy (T-FCS) approach needs to be considered in the future^26^. In location (B) near the contact line, the fluorescent particles have no directional velocity and the motion is characteristically random. This behavior is observed until the air bubble bursts, then the internal flow in the optical plane 500 nm above the substrate become the same as the droplets without an air bubble.

**Figure 5:**
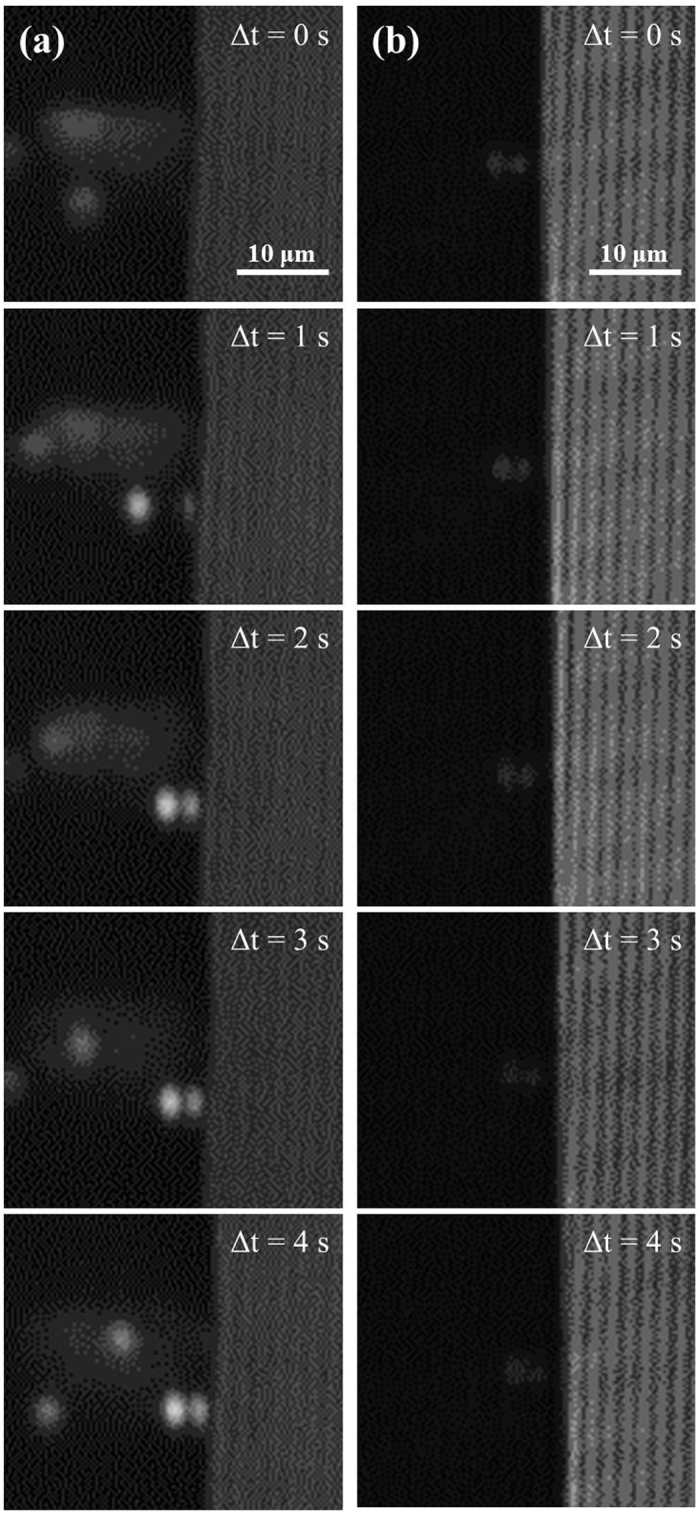
The comparison of particle motion before injecting air bubble (a) and in the presence of air bubble (b).

**Figure 6:**
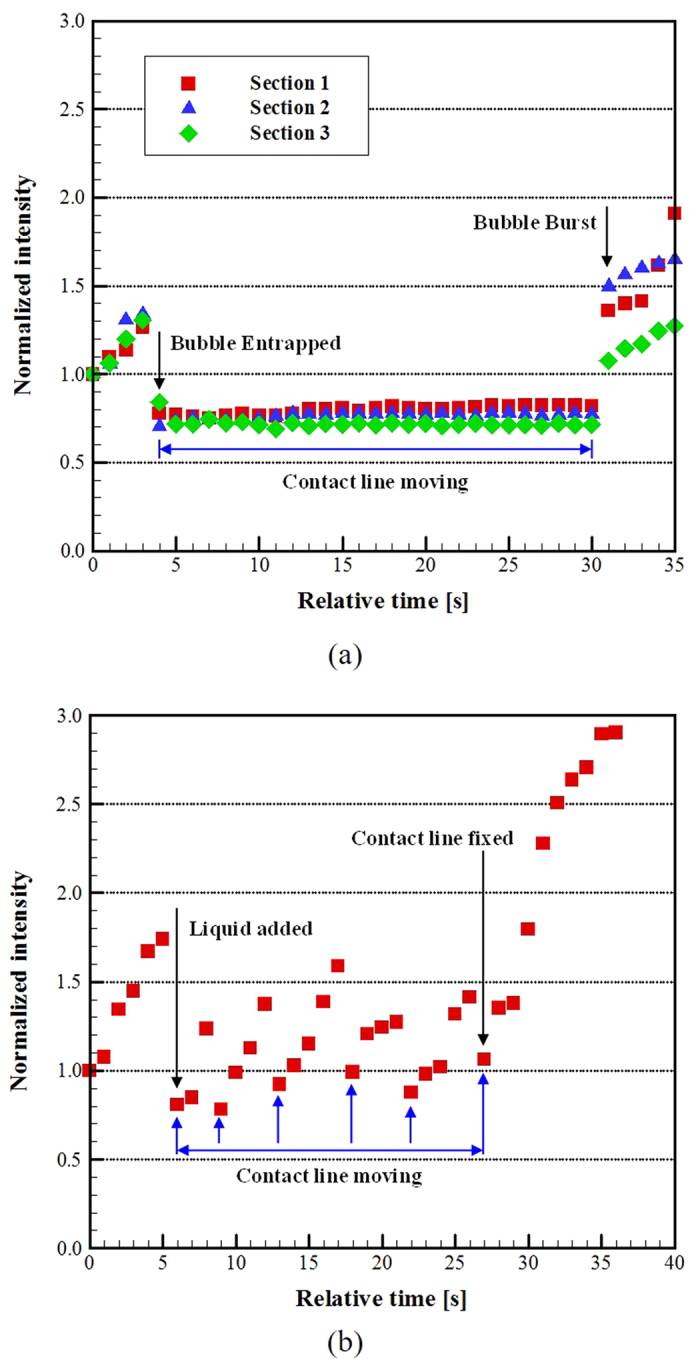
Normalized fluorescent particle intensities in the contact line region of the droplet during evaporation. (**a**) Intensity variation following air bubble injection. Sections 1, 2, and 3 in Fig. 6 represent the dashed rectangular regions shown in Fig. 4B. (**b**) Intensity variation following the injection of the test liquid into the droplet. The images are taken in the same relative location as shown in Fig. 4(B). Fluorescent intensity is integrated over 20 × 20 pixel (4.5 μm × 4.5 μm) sections and normalized using the first image of the sequence.

The flow into the contact line region during evaporation of a droplet without an air bubble results in an accumulation of fluorescent particles resulting in a continual increase in fluorescent intensity as shown in Fig. 5(a). The rate of intensity increase may be used as a diagnostic of flow without tracking individual particles. The fluorescent intensity is normalized using the image taken at approximately four seconds prior to the injection of the air bubble. Figure 6(a) shows normalized intensities of fluorescent particles in the contact line region of the droplet with respect to time sequence of images shown in Fig. 4, which are recorded during the 10 to 25 second interval relative to the beginning of the drop deposition (0 second). The contact line was maintained in the field of view as seen in the Fig. 4(B) sequence. The field of view over which the intensity was integrated was 4.5 μm by 4.5 μm. The image intensity was measured at three different sections along the contact line; each section being 20 μm along the contact line from the previous section. The same rate of intensity increase is observed in each section prior to the injection of the air bubble. Once the air bubble in injected, the intensity in each region decreased to approximately the same relative value and remained constant until the bubble burst. According to Fig. 5(b), the evaporation-driven flow seems to be stopped during the present of air bubble. At which time the intensity in each section began to increase again.

One possibility for this observation is that the radial displacement of the contact line due to the injection of the air bubble results in a material surface displacement that brings liquid from the top of the droplet into the contact line region^27^; thereby suppressing the evaporative-driven flow. After the air bubble is injected, the contact line is displaced radially in a stick-slip motion occurring on intervals of 4 to 5 seconds. The magnitude of the displacement is approximately 1.25 μm per second.

It is possible that the material surface displacement could suppress the flow into the contact line region. Thus, this study included a series of tests to determine if there was any effect due to material surface displacement. The experiments were repeated using an injection of 2 μl of liquid instead of air. Two different kinds of liquids were applied into the liquid droplet. One liquid was the same as the original droplet (0.10 vol.% Al_2_O_3_, 0.008 vol.% fluorescent particles). The second liquid was pure DI-water. Both tests showed similar results (Fig. 5(b)), opposite of that observed with the injection of the air bubble. During the radial displacement of the contact line, the intensity decreased sharply to a normalized intensity comparable to that observed for droplets with the air bubble. However, once the contact line stopped moving, the fluorescent intensity immediately began to increase indicating a flow into the contact line region. The process was repeated for each stick-slip event. The increase in intensity during intervals when the contact line was stationary was not observed when an air bubble was present as indicated in Fig. 6(a).

The air bubble remains stable for approximately 1 minute. During this time, the rate of evaporation appears to be slower, which is supported by the observation of limited flow into the contact line region. However, once the air bubble bursts, the time for the droplet to completely evaporate is decreased by approximately 5% relative to the non-bubble droplets. The increase in contact line length (increased droplet diameter) following the air bubble injection results in an overall higher evaporation rate as compared to the droplet without the air bubble.

**Figure 7:**
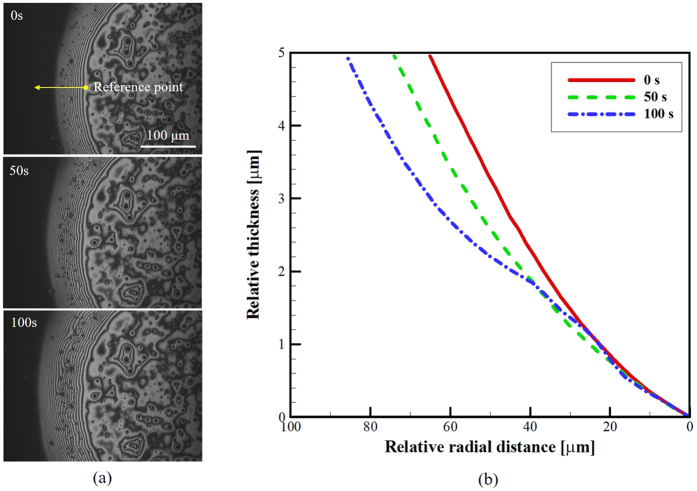
(**a**) The time-dependent fringe patterns changes at the fixed view section. Time is relative to the injection of the air bubble. (**b**) Relative elevation of the analyzed fringe patterns beneath the air bubble. The zero of relative radial distance is defined the location of the first showing fringe pattern most near the center of the droplet marked in first image, excluding chaotic shape of fringe patterns. Total numbers of fringes are the same in each time lapse.

Another possibility for the observed changes in internal flow near the substrate for droplets with and without an air bubble could be the evolution of the bubble shape. After injection of the air bubble, buoyancy would result in drainage from the liquid film defining the bubble. Upward motion of the bubble would result in radially inward flows beneath the bubble. The outward movement of the widening fringe patterns, seen in Fig. 7(a) with respect to time, represents flattening of the bubble during the stabilization process. The corresponding upward motion of the bubble due to this buoyancy effect would result in radially inward flows beneath the bubble. Radially inward flows are observed with an air bubble that is flattening against the substrate. This phenomenon can be observed in Fig. 7.

The fringe evolution beneath the air bubble (location A in Fig. 4) is shown in Fig. 7(a). As time passes, the fringes increase in size indicating the air bubble is decreasing in curvature in this location. The relative thickness of the liquid film beneath the air bubble, or alternatively the elevation of the liquid-gas interface near the substrate, is determined from analysis of the fringe patterns. The relative difference of elevation between constructive and destructive interference fringes can be calculated as *mλ*/2*n* (m = 0, 1, 2,…), where *λ* is the wave length of the illumination and *n* is the refractive index of the liquid (*λ* = 488 nm; *n* = 1.369). The time evolution of these fringes is illustrated in Fig. 7(b) which indicates a gradual decrease in elevation of the liquid-air interface beneath the air bubble. This observation is counter-intuitive relative to the viscous stresses generated by the radially inward flow.

**Figure 8:**
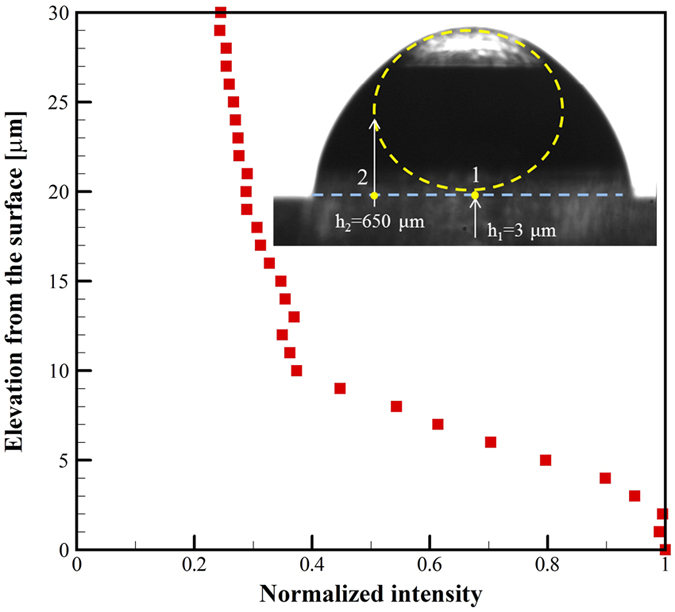
Intensity variation for 4.3 μm optical slices (20× magnification, 360 μm by 275 μm field of view) measured at 1 μm increments from the substrate at location 1 shown.

The absolute elevation of the liquid-air interface at the center of the air bubble was estimated by measuring the reflectance intensity of 20× magnification images (optical slice thickness of 4.3 μm) at 1 μm increments from the substrate shown in Fig. 8. To obtain the optical slices at different elevations, the objective lens was moved at 1-second intervals. The relative intensity of each optical slice focal plane is shown in Fig. 8. The average relative image intensity remains unchanged up to approximately 3 μm then begins to gradually decrease as the optical slice includes an increasing portion of the liquid-air interface. At approximately 10 μm above the substrate, the average relative intensity variation remains nearly constant. There is some decrease in intensity associated with reflection of light from the air-liquid and liquid-solid interfaces. Nevertheless, the optical slice can be considered to be within the air bubble at these elevations. Based on the relative intensities, the center of the bubble is estimated to be at 3 μm above the substrate and 180 μm radial position of the elevation is approximately 10 μm, which corresponds to the fringe analysis shown in Fig. 7. In a similar manner, the elevation between liquid and bubble at equatorial position of the air bubble (location 2 in Fig. 8) is approximately 650 μm.

### Concluding Remarks

Internal flow and interface shape of an evaporating droplet with an entrapped air bubble were observed using swept-field confocal microscopy. Particle motion, fluorescent intensity, and fringe analysis were used to assess the effect of the air bubble on evaporation processes. The presence of the air bubble suppresses flow into the contact line region and generates a radially inward flow near the center of the droplet at the substrate. Injection of the air bubble results in a stick-slip contact line motion. This motion naturally suppresses evaporation-driven flow into the contact line. However, measurements of fluorescent intensity and fringe analysis of the bubble shape indicate that other physical mechanisms are responsible.

**Figure 9:**
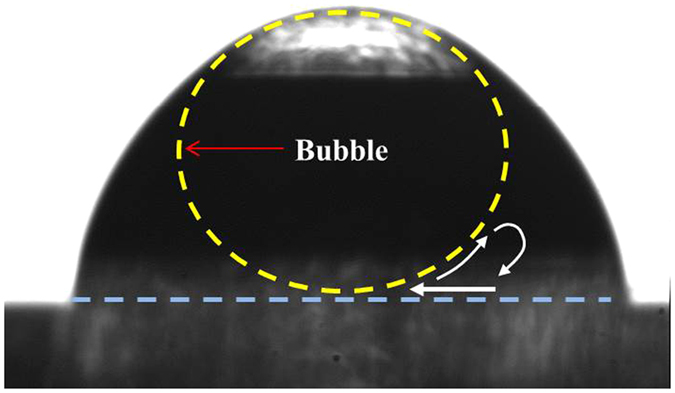
Observed internal flows for the case of an entrapped air bubble inside the droplet.

When the optical slices were moved incrementally upward from the substrate, the particle trajectories indicated a surface flow along the gas-liquid interface towards the bubble equator as shown in Fig. 9. This liquid flow recirculates before reaching the equatorial plane of the bubble, yet the bulk liquid in the vicinity of the contact line region remains quiescent. In this situation, the evaporation-driven flow could be stopped and reverse flow can generate near the center of the droplet beneath the droplet. Moreover, the presence of the air bubble, even though short-lived, affects the evaporation-driven flow. The air bubble presence suppresses flow in the contact line region and induces flow towards the center bottom of the droplet. The presence of the air bubble is also accompanied by an observed absence of particle deposition in the contact line region. We hypothesize that these two observations are interrelated, though the experimental technique was established to study internal flow and not particle deposition. The effect of the air bubble on particle deposition rates and patterns in the contact line region is an interesting opportunity for follow-on studies.

## Additional Information

**How to cite this article**: Shin, D. H. *et al*. Observations of internal flow inside an evaporating nanofluid sessile droplet in the presence of an entrapped air bubble. *Sci. Rep*. **6**, 32767; doi: 10.1038/srep32767 (2016).

## Figures and Tables

**Figure 1:**
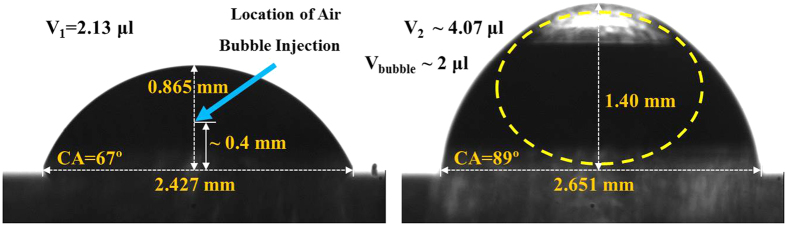
Profile images of a 2 μl droplet without (left) and with (right) a 2 μl air bubble.

**Table 1:** The various conditions for a possibility to entrap an air bubble inside the droplet.

Liquid	Parameter	Parameter Range	Bubble Stability
Pure liquid	DI-water	—	Unstable
Nanoparticle concentration	50 nm Al_2_O_3_	Low vol.% (0.01, 0.05)	Unstable
		High vol.% (0.10)	Stable
Liquid-to-air volume ratio (2 μl air bubble)	50 nm Al_2_O_3_	Volume ratio >2.5	Unstable
	0.10 vol.%	1 ≤ Volume ratio ≤ 2.5	Stable
Surfactant	Span^®^ 80	Same molecular weight with that of 0.10 vol.% nanofluid case	Unstable
Fluid viscosity	Glycerol solution	Low mass% (10)	Unstable
		High mass% (30)	Unstable
